# The Diseases of the Nervous System, a Text-Book for Physicians and Students

**Published:** 1893-08

**Authors:** 


THE DISEASES OF THE NERVOUS SYSTEM, A TEXT-BOOK FOR PHYSICIANS AND STUDENTS, by Ludwig Hirt, M. D., Professor at the University of Breslan, Translated by August Hoch, M. D., assisted by Frank R. Smith, A. M., (Cantab) M. D., Assistant Physicians to the Johns Hopkins Hospital, with an introduction by Wm. Oslin, M. D., F. R. C. P., with 176 illustrations ; Published by D. Appleton & Co., N. Y., 1893.

Rarely do we find a translation of any scientific subject that just expresses the authors intent and purpose, but such do we find in this translation of Dr. Ludwig Hirt's work.

The original approaches as near an authority as the progressive art of medicine will allow, and the author deserves the unanimous thanks of the profession of medicine. His effort has surely been crowned with success, and the translators deserve equally as much praise in their capacity.

The work is exhaustive, true and original. The classification is fine, and his wonderful candor in prognosis unsurpassed. Much medical and more moral truth is contained within its pages.

H. H.

---

All physicians of experience agree that no class of cases are so difficult to treat satisfactorily as those suffering from prolapsus of the internal organs, but it is with pleasure we call attention of the profession to the merits of The Natural Body Brace, advertised in this issue as a means to bring about the most satisfactory results to both the physician and patient.

